# Respiration Subservient to Nutrition; a Thesis Presented to the Medical Faculty of Harvard University, March, 1850

**Published:** 1853-07

**Authors:** 


***Respiration subservient to Nutrition; a Thesis presented to the Medical Faculty of Harvard University, March,*** **1850. By Dr. E. Leigh. Boston. Ticknor, Reed & Fields, 1853.**

Dr. Leigh, is, as we have already seen, fond of speculating and reasoning upon the facts of medicine. The little pamphlet before us is a specimen of his method of speculation. He has no new facts to bring forward, but he closely and studiously examines old ones, and points out the way in which he thinks new observations ought to walk.

In this *brochure,* he has pointed out several facts, which show that the absorption of oxygen must be designed for some other purpose than the mere combustion of carbon. He thinks it has direct reference to nutrition, that it is, in short, an aërial *food,* so that the old fable of the chameleon living on air, was not wholly an idle dream. We regard this view of the matter as highly probable, especially when it is considered that the tissues of the body contain more oxygen than the food from which they are formed, a fact thoroughly established by recent chemical researches.

